# Facial Emotion Recognition and Expression in Parkinson’s Disease: An Emotional Mirror Mechanism?

**DOI:** 10.1371/journal.pone.0169110

**Published:** 2017-01-09

**Authors:** Lucia Ricciardi, Federica Visco-Comandini, Roberto Erro, Francesca Morgante, Matteo Bologna, Alfonso Fasano, Diego Ricciardi, Mark J. Edwards, James Kilner

**Affiliations:** 1 Neurosciences Research Centre, Cardiovascular and Cell Sciences Research Institute, St George's University of London, London, United Kingdom; 2 Sobell Department of Motor Neuroscience and Movement Disorders, UCL Institute of Neurology, University College London, London, United Kingdom; 3 Department of Clinical and Experimental Medicine, University of Messina, Messina, Italy; 4 Department of Neurology and Psychiatry, Sapienza University of Rome, Rome, Italy; 5 Neuromed Institute IRCCS, Pozzilli, Isernia, Italy; 6 Morton and Gloria Shulman Movement Disorders Clinic and the Edmond J. Safra Program in Parkinson’s Disease, Toronto Western Hospital, UHN, Division of Neurology, University of Toronto, Toronto, Ontario, Canada; 7 Department of Geriatry, Catholic University of Sacred Heart, Rome, Italy; Aristotle University Of Thessaloniki Faculty of Forestry and Natural Environment, GREECE

## Abstract

**Background and aim:**

Parkinson’s disease (PD) patients have impairment of facial expressivity (hypomimia) and difficulties in interpreting the emotional facial expressions produced by others, especially for aversive emotions. We aimed to evaluate the ability to produce facial emotional expressions and to recognize facial emotional expressions produced by others in a group of PD patients and a group of healthy participants in order to explore the relationship between these two abilities and any differences between the two groups of participants.

**Methods:**

Twenty non-demented, non-depressed PD patients and twenty healthy participants (HC) matched for demographic characteristics were studied. The ability of recognizing emotional facial expressions was assessed with the Ekman 60-faces test (Emotion recognition task). Participants were video-recorded while posing facial expressions of 6 primary emotions (happiness, sadness, surprise, disgust, fear and anger). The most expressive pictures for each emotion were derived from the videos. Ten healthy raters were asked to look at the pictures displayed on a computer-screen in pseudo-random fashion and to identify the emotional label in a six-forced-choice response format (Emotion expressivity task). Reaction time (RT) and accuracy of responses were recorded. At the end of each trial the participant was asked to rate his/her confidence in his/her perceived accuracy of response.

**Results:**

For emotion recognition, PD reported lower score than HC for Ekman total score (p<0.001), and for single emotions sub-scores happiness, fear, anger, sadness (p<0.01) and surprise (p = 0.02). In the facial emotion expressivity task, PD and HC significantly differed in the total score (p = 0.05) and in the sub-scores for happiness, sadness, anger (all p<0.001). RT and the level of confidence showed significant differences between PD and HC for the same emotions. There was a significant positive correlation between the emotion facial recognition and expressivity in both groups; the correlation was even stronger when ranking emotions from the best recognized to the worst (R = 0.75, p = 0.004).

**Conclusions:**

PD patients showed difficulties in recognizing emotional facial expressions produced by others and in posing facial emotional expressions compared to healthy subjects. The linear correlation between recognition and expression in both experimental groups suggests that the two mechanisms share a common system, which could be deteriorated in patients with PD. These results open new clinical and rehabilitation perspectives.

## Introduction

Facial expression of emotion is a key tool for communicating with others. Likewise, our ability to interpret the emotional facial expression of others is key to successfully understanding what others are communicating to us. As would be expected given the complexity of producing or interpreting emotional facial expression, distributed brain networks have been proposed as being involved, including cortical and sub-cortical regions.

One important and unresolved question is: to what extent does the facility for emotional facial expression overlap with the facility for interpretation of the emotional facial expression of others? The concept of “mirroring” of observed action by activation of the sensorimotor network of the observer is widely discussed in the motor control literature, but does this have relevance for the expression and understanding of facial emotion? In an fMRI study of healthy participants, largely overlapping patterns of activation were noted for both observation and imitation of facial emotional expressions, including the premotor face area, the pars opercularis of the inferior frontal gyrus, the superior temporal sulcus, the insula and the amygdala [1]. These observed activations fit with previous works investigating the neural correlates of emotional expression and interpretation of the emotional facial expression of others.

However, there are a number of outstanding issues with the current evidence in favour of a link between the mechanisms of emotion expression production and recognition. Firstly, the demonstration of overlapping activation patterns in two different tasks does not necessarily imply co-dependence of behavioural abilities linked to these activations. Secondly, there is evidence that different emotions (and their recognition) may involve different brain regions. A recent meta-analysis of neuroimaging studies in 1600 individuals has suggested that basic emotions are implemented by neural systems that are at least partially separable, although they may not be represented by entirely distinct neural circuits [2]. Happy and fearful faces activate the amygdala bilaterally, sad faces the right amygdala only, disgust seems to activate preferentially the anterior insula [3]); fear seems to preferentially activate the amygdala [4].

Patients with Parkinson’s disease (PD) provide an interesting model to address these issues directly. Indeed, patients with PD are known to have impairment of both spontaneous and posed facial expressivity [5–12]. With regard to recognition of emotional facial expression, a recent meta-analysis evaluating only behavioural studies found that individuals with PD were more impaired than healthy individuals in the recognition of negative emotions (anger, disgust, fear, and sadness) than those of relatively positive emotions (happiness, surprise) [13].

The aim of the present study was to evaluate the ability to produce facial emotional expressions and to recognize facial emotional expressions produced by others in a group of non-demented, non-depressed PD patients and a group of matched healthy participants. We were specifically interested in exploring the relationship between the participants’ ability to express and their ability to recognize facial emotional expressions and any differences between the two groups of participants.

## Methods

### Subjects

Twenty PD patients were included in the study. Inclusion criteria were: a diagnosis of PD according to UK Brain Bank criteria [14], treatment and clinical condition stable for at least 4 weeks prior to the study; absence of significant cognitive deficits or score < 24 at the Mini Mental State Examination (MMSE) [15]; absence of depression [diagnosed according to both the DMS-IV TR criteria and Beck Depression Inventory (BDI) [16]score ≥ 17] or other psychiatric or neurological illnesses. Twenty healthy controls (HC) matched for age and gender were enrolled and served as control group; they also underwent cognitive and psychiatric testing to rule out cognitive impairment and depression. Demographic data of all the study participants were collected; for patients, clinical information such as disease duration, and dopaminergic therapy expressed in terms of Levodopa Equivalent Daily Dose (LEDD) [17] were included. Patients underwent a clinical assessment of motor impairment by means of the Unified Parkinson’s disease rating scale (UPDRS) section III [18], the total score was taken into account as well as the sub-score of item 19 evaluating facial expressions. Demographical and clinical data of PD and HC are presented in Table 1. All participants agreed to participate in the study and have given written informed consent (as outlined in PLOS consent form) to publish these case details. UCL institutional review board approved the study protocol.

**Table 1:** Demographic, clinical and emotion recognition (Ekman 60 Faces Test) data of the study populations.

	PD (n = 20)	HC (n = 20)	*p-*value
***Gender***	12 F	11 F	0.7
***Age (years)***	69.3±6.6	65.9±6.4	0.1
Education level* ***(years)***	15.1±5.1	20.5±4.9	0.7
***MMSE***	28.3±1.4	29.7±0.8	0.4
***BDI***	9.7±2.7	8.9±4.9	0.5
***UPDRS-III***	21.8±8.7	------	------
***UPDRS-III item 19***	1.8±0.7	------	------
***Disease duration (years)***	7.3±4.1	------	------
***LEDD (mg)***	612.5±370.5	-------	------
***Ekman-total score***	**44.5±7.3**	**52±4.1**	**<0.001**
***Ekman-happiness***	**9.6±0.7**	**10**	**0.02**
***Ekman-sadness***	**7±2.7**	**8.7±1.3**	**0.03**
***Ekman-anger***	**7.4±1.6**	**8.9±1.14**	**0.003**
***Ekman-disgust***	8.2±1.9	9±0.9	0.12
***Ekman-fear***	**4±2.7**	**6.5±2.8**	**0.01**
***Ekman-surprise***	**8.2±2.3**	**9.5±0.4**	**0.01**

Values are expressed as mean±standard deviation. Abbreviations: BDI: Beck’s Depression Inventory; F: Female; HC: healthy subjects; LEDD: levodopa equivalent daily dose; MMSE: Mini Mental State Examination; PD: Parkinson’s disease; UPDRS: Unified Parkinson’s disease Rating scale.

* total number of years spent at school (from primary school to university).

### Procedure

**Fig 1:**
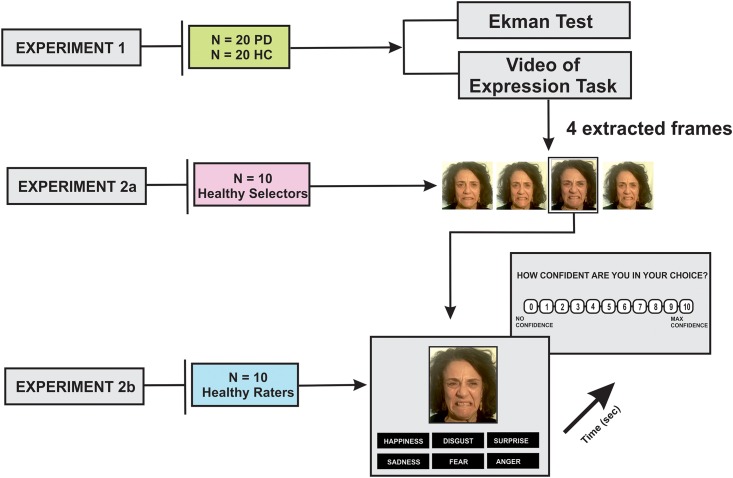
Experimental design displaying the main 3 experiments. In experiment 1, Parkinson’s disease (PD) and Healthy Controls (HC) subjects performed an emotion recognition task and a facial expressivity task (which was videotaped). In experiment 2a, ten healthy subjects selected the most expressing frames extracted from the videos for each emotion and for each subject. In experiment 2b, ten healthy raters (different from those employed in experiment 2a) judged PD and HC expressivity. At the end of each trial, the participant was asked to rate his/her level of confidence in their choice clicking the mouse on a visual analogic scale on the screen, where “max confidence” was 10 “no confidence at all” was 0. Reaction time (RT; in sec) and accuracy of responses were recorded.

The experimental procedure included three main experiments (experiment #1, experiment #2a, experiment #2b) which are summarized in Fig 1.

#### Experiment #1: Facial recognition task and facial expression video protocol

The experimental procedure was conducted in a soft-lighted, sound-attenuated room. All patients evaluations were performed after taking the first dose of dopaminergic medications, once they reached their best motor state (“ON” medication).

All subjects (PD and HC) underwent a standardized video to record facial expression, including face, hair, and shoulders, as described by Ekman and Friesen [19]. They were asked to pose facial expressions representing six distinct emotions (happiness, sadness, surprise, anger, disgust, and fear) [20].

In the facial recognition task, PD and HC were assessed by means of the Ekman 60 Faces test [21]. Pictures of 10 actors’ faces expressing the six basic emotions (happiness, sadness, surprise, anger, disgust, fear) were selected from a set of validated pictures, i.e. the Pictures of Facial Affect [21] and randomly presented on a computer screen, one at a time. Participants were asked to identify the emotional label in a six-forced-choice response format (alternatives: happy, sad, surprise, angry, disgust, fear). Response time was unlimited, but participants were encouraged to respond as quickly as possible. The test yields a score out of a maximum of 60 correct for recognition of all six emotions, or scores out of 10 for recognition of each basic emotion. In order to ensure participants’ understanding of the task, the emotional labels were confirmed prior to testing by asking the subjects to describe an example of scenario for each emotion (“Name a situation when you felt happiness, fear, etc…).

#### Experiment #2a: Selection of the most expressive frames

To assess the expression of emotions, it was required to have still images of each participant (HC and PD) expressing the six basic emotions. That is, we extracted from the videos still images of each PD and HC subject, for each of the six emotions tested. For each of the video segments, a window of 4 s was selected that showed the expression of the intended emotion. The total duration of each single scene showing the best facial emotion expression was calculated and the duration of the video was divided into four sections of equal length. Finally, one still image from each section was then selected that was deemed to best express the intended emotion. This produced 4 images per participant per emotion to be used in experiment #2a designed to select which of these 4 images best expressed the intended emotion in an unbiased way.

To this aim, we programmed a computerized task, in which the 4 different pictures selected for each participant and for each emotion were presented on the computer screen (colour images, 6.3 × 4.5 cm in size). Ten healthy selectors (7 females/3 males, mean age 33 ± 3.0 years) were asked to select the most expressive one out of the four frames (Fig 1B). Participants made their decision by selecting with the mouse the chosen frame. For each emotion the procedure was repeated 4 times with each frame appearing always in a different position on the screen to avoid any spatial bias. To determine which frame best expressed the emotion, a weighted average of the frequency of expressivity rank was calculated across repetitions and across the ten participants. Frames ranked 1^st^ were assigned the weight 2, frames ranked 2^nd^ were assigned the weight 1, frames ranked 3^rd^ were assigned the weight 0.5 and frames ranked 4^th^ were assigned the weight 0. The frame with the highest average weighted ranking was chosen as the most expressive of the four. When this analysis failed to give a single choice among the four (19/120 cases), we selected frame ranked first the most often across participants.

#### Experiment #2b: Facial expressiveness evaluation

Ten healthy raters (7 females/3 males, mean age 29 ± 2.0 years), different from those recruited for experiment #2a, were enrolled among clinical and research fellows at the University College of London. Subjects were excluded if they presented impairment of facial emotion recognition as assessed by means of the Ekman 60 Faces test prior to the main experiment (see above for details on Ekman 60 Faces test).

Each rater was seated in front of a high-resolution computer monitor at a visual distance of approximately 60 cm. The previously selected frames (experiment #2a) of 20 PD patients and 20 HC, posing the 6 facial emotions, were presented on the screen in random fashion. Six replications for each picture were included, for a total of 1440 trials (6 emotions x 6 trials x 40 posing subjects). Participants were asked to identify the emotional label in a six-forced-choice response format (alternatives: happyness, sadness, surprise, anger, disgust, fear), and were instructed to click the mouse on the chosen label displayed on the bottom of the picture. It has been assigned "0" for uncorrected response and "1" for corrected responses. The score range for each emotion (relative score) ranged from 0 to 6 (facial expressivity score). Reaction time (RT; sec) and accuracy of responses were also recorded. At the end of each trial, each subject was asked to rate his/her confidence in their perceived accuracy of response. This confidence judgement was made asking the participant to click the mouse on a visual analogic scale that was 10 cm long, displayed on the screen. One end was marked “max confidence” (10), while the other end was labelled “no confidence at all” (0) (Fig 1C).

All experiments were run with MatLab software (version 2014b, Mathworks MA, USA). In order to avoid fatigue and make the task more feasible, we divided it in 4 different blocks, each one consisting of 360 trials. Participants performed the task in two consecutive days, 2 blocks each day.

### Statistical analysis

Two-way analysis of variance (ANOVA) was applied to the Ekman total score with a 2x1 design (within-subjects factor: Ekman score; between-subjects factor: PD and HC). The Ekman sub-scores were included in a 2x6 mixed-design ANOVA, with *“group”* (two levels: PD, HC) as between-subjects factor and *“emotion”* (6 levels: happiness, sadness, anger, fear, surprise and disgust) as within-subjects factor. An analogue statistical design was performed to evaluate differences between PD and HC in the facial expressivity total score and in the sub-scores for each emotion. We performed two separates 2x6 ANOVAs with group (PD, HC) as between-subjects factor and “emotion” as between-subjects factor, for the variables reaction time (RT) and confidence level (CL). We analysed the RT and CL of the correct answers. The linear relation between RT and confidence level was analysed as measure of metacognition [22]. Conditional to significant F-values in the ANOVA, post-hoc unpaired t-tests were performed to highlight differences between groups.

Correlational analysis was run by the Pearson correlation test, in order to test possible correlations between facial emotion recognition (Ekman total score and single emotion sub-score) and facial emotion expressivity (facial expressivity total score single emotion expressivity sub-score). The first correlation analysis was aimed to probe the presence of a relationship between the ability to express the emotions and the ability to recognise the emotions. Thus, we correlated each participant’s average score for the facial recognition task with each rater’s average score for the facial expressivity task considering all emotions together. In a subsequent analysis, Pearson correlation was employed on the mean value across subjects (PD or HC) for each emotion in the facial expressivity task and for each emotion in the Ekman recognition test. Finally, in order to evaluate whether the relation between recognition and expression was emotion-dependent, we performed a correlation analysis ranking the emotions on the basis of the recognition performance by both PD and HC. Namely, for each subject we ranked the emotions from the best recognized to the worst recognized in the recognition task and we gave the same order to the emotions for the expressivity task. We considered the mean value in each group (PD and HC) for each ranked emotion for both recognition and expression and we performed the correlation analysis. A p-value ≤ 0.05 was considered significant.

## Results

### Facial emotion recognition in PD and HC

**Fig 2:**
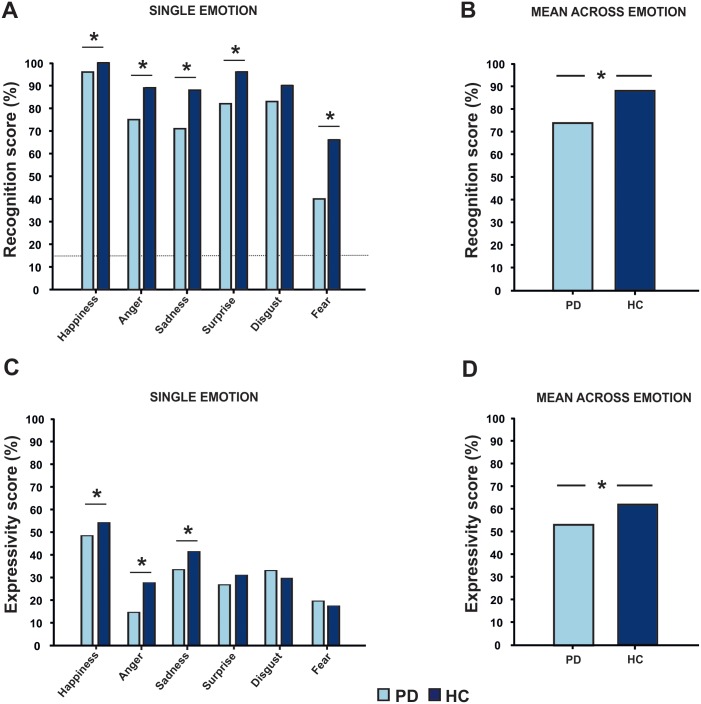
Panel A and B: Facial emotion recognition task (Ekman 60 Faces test) scores for each single emotion (A) and total score for all emotions taken together (B) for HC (dark blue) and PD group (light blue). Higher scores indicate better performance. PD patients performed worse than HC in all emotions but disgust. Dash line represents the cut-off above which participant performs better than chance (see text for details). Panel C and D: Facial expressivity task scores for HC and PD. Higher scores indicate better performance. PD patients were judged less expressive than HC for all emotions but disgust, fear and surprise. For both tasks we plotted the percentage of corrected answer (%) to facilitate the understanding of the results. Asterisks represent a statistical difference between experimental groups (p<0.05)

Factorial ANOVA revealed a significant main effect of the factors *“group”* (F_(1,228)_ = 33.84 p<0.001) (Fig 2A) and *“emotion”* (F_(5,228)_ = 28.15 p<0.001) (Fig 2B) with no significant interaction between these two factors. Post-hoc t-tests revealed a statistical difference for happiness, anger, sadness, surprise and fear (p<0.05), which were less recognized by the PD group. Despite PD patients performed significantly worse in the facial emotion recognition task, it is important to underline that they were significantly better than chance at the task in each emotion as shown in Fig 2A by the dash lines; as each subject (HC and PD) has 1/6 chance to get a right answer for each picture of the 6 emotions for 10 different actors’ pictures, the possibility that one participant can get a right answer only by chance is 16% (1:6 = X:100 [X = 100/6]) (Fig 2A and 2B).

### Facial expressivity in PD and HC

A significant effect for both the factors *“group”* (F_(1,228)_ = 3.83 p = 0.05) and *“emotion”* (F_(5,228)_ = 21.24 p<0.001) emerged at the ANOVA (Fig 2, panels C and D), without any interaction between these two factors. Post-hoc t-tests revealed that happiness, anger and sadness were less recognized by the PD group (p<0.001) compared to HC.

### Reaction time and confidence level in the facial expressivity test

**Fig 3:**
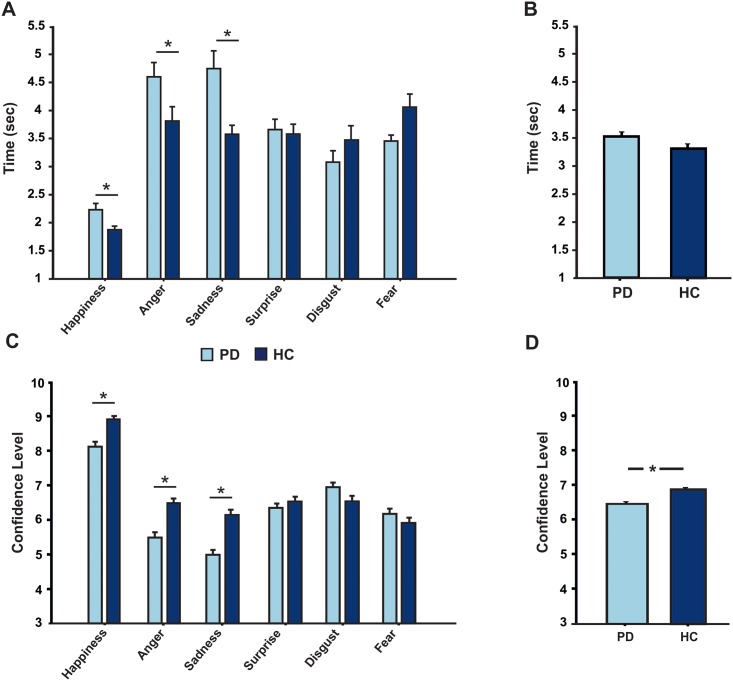
A) Reaction Time in choosing the pictures of PD (light blue) and HC (dark blue) in the expressivity task. Raters were faster in judging HC than PD pictures expressing happiness, anger and sadness. B) Level of confidence in evaluating which emotional expression was displayed in the pictures of PD (light blue) and HC (dark blue) in the expressivity task. Raters were more confident of their choices when evaluating HC’ pictures compared to PD’ s pictures for happiness, anger and sadness.

For RT, ANOVA showed a significant effect of the factor *“emotion”* (F_(5,224)_ = 8.33, p<0.001), but not of the factor “group” (Fig 3A and 3B). No interaction between these factors was present. For CL, ANOVA showed a significant effect of the factors *“emotion”* (F_(5,222)_ = 21.42, p<0.001) and *“group”* (F_(1,222)_ = 4.28, p = 0.03) (Fig 3C and 3D).

**Fig 4:**
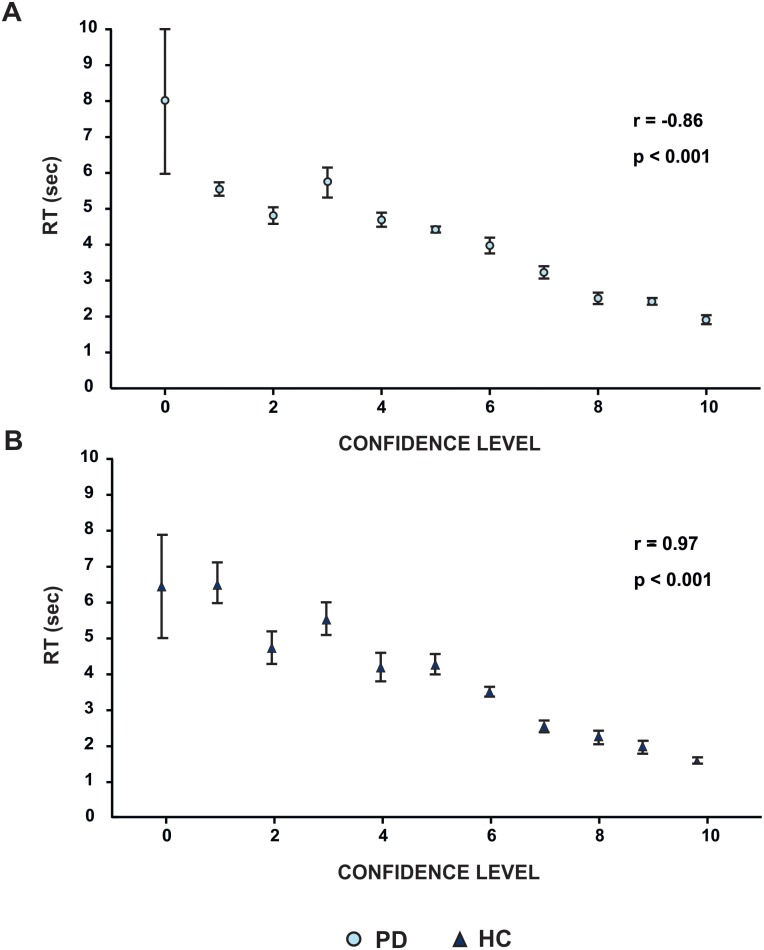
Correlation between raters’ Reaction Time (RT) in choosing the pictures and their level of confidence during their own choices (CL) for both the PD (panel A) and HC (panel B) frames during the expressivity task. A positive correlation between RT (y axis) and CL (x axis) is shown.

As expected, we showed that the participants' level of confidence in their decision was correlated with their RT. The higher level of confidence they had, the shorter the reaction time in choosing the picture was. This was true for both PD (r = 0.86, p<0.001) and HC frames (r = -97, p<0.001) (Fig 4A and 4B).

### Correlation between facial emotion expressivity and facial emotion recognition

**Fig 5:**
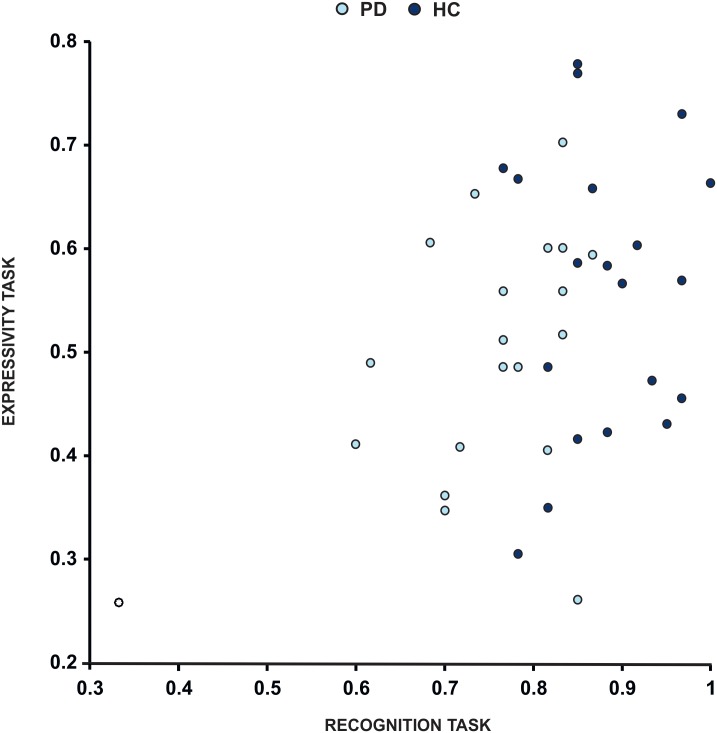
Correlation between facial recognition task scores and expressivity task scores. Each dot represents the mean value across emotions per subject in HC group (light blue) and PD group (dark blue). There is a significant positive correlation between facial recognition (x axis) and expressivity (y axis) for all subjects.

The relationship between the ability to express the emotion and the ability to recognise the emotion was significant when considering all participants from the two groups together (r = 0.39, p = 0.01); yet, when looking at the correlation at each single group level, only the PD group showed a significant correlation (r = 0.48, p = 0.02) (Fig 5). When considering single emotions, a significant positive correlation between recognition and expressivity scores was revealed only for surprise (r = 0.55, p = 0.01) in the PD group and a tendency toward statistical significance was also observed for disgust (r = 0.4, p = 0.06) for the same group.

**Fig 6:**
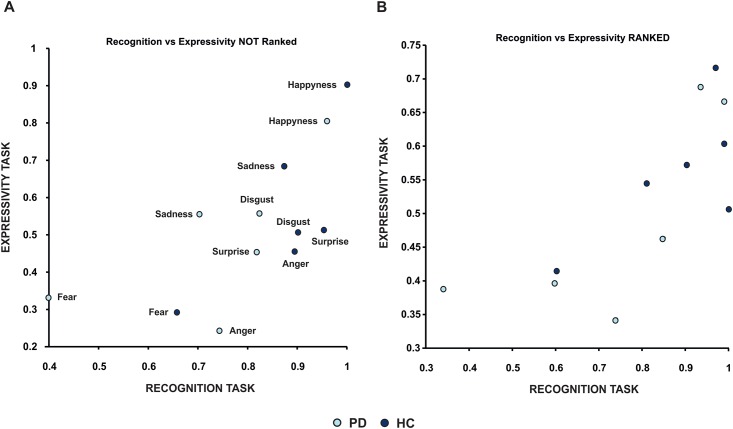
Correlations between facial emotion recognition and expression per single emotion. Each dot represents the mean value across subjects per each emotion for both HC (light blue) and PD (dark blue). There is a significant positive correlation between recognition (x axis) and expression (y axis) in both groups (panel A, each emotion is displayed next to the corresponding dot). The correlation is even stronger when ranking emotions from the best recognized to the worst (Panel B, see text for details).

To further investigate the relationship between emotional recognition and expressivity, we calculated the grand average of the facial expressivity score and the recognition score for each emotion in the PD and HC groups and we performed a correlation analysis between these two measures. Results revealed that when treating PD and HC groups as independent there was a significant correlation between emotion expression and emotion recognition (r = 0.67, p = 0.01) (Fig 6A). This analysis (although significant) assumes that all participants performed equally for each emotion. If there were truly a link between emotion expression and recognition then one would predict that the emotion that any individual was worse at recognising they would also be bad at expressing. To test this we ranked the order of the emotions for each individual in the two groups (PD and HC) from the best recognized to the worst and we modified accordingly the order of the emotions for the expressivity task. We calculated this for each subject and we calculated the mean value for each ranked emotion. We found an even greater significant correlation (r = 0.75, p = 0.004, Fig 6B), suggesting that the relation between expression and recognition is not emotion dependent.

## Discussion

We demonstrated a relationship between the ability to express facial emotions and the ability to recognize facial emotions expressed by others. Interestingly, this relationship was not emotion-specific, as each subject differed from the others in terms of emotion affected both for its expression and its recognition. That is, in both healthy subjects and PD patients, better performance in a task of execution of facial emotional expression was related to a better performance in a well-validated task of facial emotion recognition. Nevertheless, PD patients performed worse in both tasks than healthy controls.

The motivation for this study was to explore the relationship between facial emotional expression and recognition of these expressed emotions in others. While functional imaging studies have suggested overlapping areas of activation during tasks of emotional expression and emotional recognition, this falls short of evidence for co-dependence of these faculties. Here, by evaluating participants on both an execution and recognition task, we were able to assess within subjects the relationship between performance on both tasks, and found a strong correlation between them. Thus, we provide strong additional evidence for a shared substrate for these tasks. We found a strong relationship irrespective of the type of emotion, suggesting an involvement of an overall unified system, instead of different neural systems for specific emotions.

We evaluated both PD patients and HC and therefore provide additional data regarding emotional expression and recognition in Parkinson’s disease. First, although performance in tasks of emotional expression and recognition was impaired in PD patients compared to controls in line with previous data [5–12], the correlation between performance on the two tasks found in healthy subjects and in PD patients is a novelty provided by our study. This adds weight to our suggestion that both tasks share a common substrate that is disrupted in patients with PD. Second, our data in PD patients addresses some areas of conflicting data in the literature. An extensive literature has evaluated emotional recognition ability in PD patients with conflicting results likely due to confounding factors (e.g. depression and cognitive deficits) and methodological differences [23,24]. In a recent meta-analysis, evaluating only behavioural studies, Gray and Tickle-Degnen suggested that individuals with PD disclosed a lower recognition score for negative emotions (anger, disgust, fear, and sadness) than for relatively positive emotions (happiness, surprise) [13]. Here we found a more extensive deficit in recognizing facial expressions, including positive and negative emotions, when a well-validated task (Ekman 60-faces test) is administered and when confounding factors such as depression and cognitive impairment are controlled for.

A previous study has found a correlation between a task of emotional facial imagery and tasks of emotional facial expression and recognition [8]. This study provides some supportive evidence for our contention that tasks of emotional facial expression and recognition are co-dependent, though in this previous study different emotions were not assessed separately and this correlation was not tested in healthy participants, limiting the scope of the conclusions.

Third, our data provide some support for “simulation” or "shared substrates" models of emotion recognition, which are based on the assumption that the ability to recognize the emotions expressed by other individuals relies on processes that internally simulate the same emotional state in ourselves [25]. These models have clear overlap with the suggested “mirror neuron” system related to voluntary movement. Mirror neurons were first identified in the ventral premotor cortex (area F5) of the macaque [26,27], subsequently they have been demonstrated in human homolog of area F5, the pars opercularis in the inferior frontal gyrus [28]. The property of these neurons is that they show activation triggered both by observation of an action and execution of that same action [29].

In an fMRI study on healthy participants, largely overlapping regions were activated by both observation and imitation of facial emotional expressions. Areas with common activation in the two tasks included the premotor face area, the dorsal sector of pars opercularis of the inferior frontal gyrus, the superior temporal sulcus, the insula, and the amygdala [1]. It has been suggested that in this network the insula may act as an interface between the frontal component of the mirror neuron system and the limbic system, “translating” the observed or posed facial emotional expression into its internally represented emotional meaning [1].

Our data are in keeping with the idea that a system, similar to the mirror system for goal-directed actions, supports both the action of facial emotional expression and the ability to correctly identify the facial emotional expression of others. In contrast to other studies, our data support the suggestion that this shared system is emotion-independent, and that while specific brain areas may be more involved in specific emotions (e.g. amygdala for fear [4,30] or insula for disgust [3], there is a broader mechanism for emotional “sense”. The hypothesis of a shared system for execution and recognition of facial emotional expression is supported by previous studies. Work in healthy participants has shown that “blocking” facial movements while performing a task of facial emotion recognition leads to a lower accuracy of performance [31]. In addition, fMRI studies on patients affected by autism, who have an impairment in recognizing facial emotions [32], have demonstrated a lack of activity in the “mirror area” in the pars opercularis when observing facial emotional expressions [33]. It has also been suggested that patients with autism have difficulties interpreting the expressions of other individuals as well as expressing emotions in a way others can understand [34]. Finally, patients with Huntington's disease (HD), have both an impairment of recognition and expression of facial emotions, and a role for the striatum in mediating facial emotional expression and recognition since performance in both tasks was correlated with the degree of striatal atrophy in these patients [35].

An important and seemingly contradictory study in patients with Moebius syndrome, a disorder of congenital bilateral facial paralysis, found no emotion recognition impairment in these patients, questioning the role of emotional facial expression recognition of emotional expression in others [36]. However, the congenital nature of this disorder raises the question as to whether compensatory mechanisms for emotion recognition have developed. The assessment of (admittedly rare) patients with acquired bilateral facial nerve palsy would certainly be of interest.

We acknowledge limitations of our study. We did not have concurrent functional imaging, which does not allow us to correlate our behavioural results with patterns of brain activation for comparison with previous studies. We did not evaluate spontaneous facial expression but rather focussed on voluntarily posed expressions. Finally, although we excluded patients with overt cognitive impairment (by means of MMSE evaluation), an extensive neuropsychological evaluation is lacking and future studies are encouraged to rule out any associated deficit in specific cognitive domains.

In conclusion, our data demonstrate a strong relationship between the execution and recognition of facial emotional expression in both healthy subjects and people with PD. Patients with PD performed overall poorer on both tasks. Although our data allow us only to speculate on the possible mechanisms underlying this process, they are consistent with an emotional mirror neuron mechanism.
